# A comparison of rigid tape and exercise, elastic tape and exercise and exercise alone on pain and lower limb function in individuals with exercise related leg pain: a randomised controlled trial

**DOI:** 10.1186/1471-2474-15-328

**Published:** 2014-10-02

**Authors:** Melinda M Franettovich Smith, Sonia S Coates, Mark W Creaby

**Affiliations:** School of Physiotherapy, Australian Catholic University, Brisbane, Australia; School of Exercise Science, Australian Catholic University, Brisbane, Australia

**Keywords:** Shin pain, Medial tibial stress syndrome, Anti-pronation

## Abstract

**Background:**

Exercise related leg pain (ERLP) is a common lower limb overuse injury characterised by pain located between the knee and ankle that occurs during activity. The high incidence of the condition, subsequent interference with participation in physical activity and substantial recovery time, highlights a need for effective interventions. Whilst many interventions have been described for the management of ERLP, currently there is a lack of high quality evidence for an effective intervention for the condition.

**Methods/Design:**

A single-blinded randomised controlled clinical trial will be conducted in a community setting. Forty-five female volunteers aged between 18 and 40 years with a history of insidious onset of pain located between the knee and ankle of at least one month duration that is aggravated by weight bearing activities will be recruited for the study. Suitable participants will be randomly allocated to one of three treatment groups for the 6 week intervention period: (i) exercise only, (ii) rigid anti-pronation tape and exercise, (iii) elastic anti-pronation tape and exercise. Outcomes will be measured at baseline, 1, 2 and 6 weeks using primary outcome measures of usual and worst pain visual analogue scale and global perceived improvement. Secondary outcome measures will include Foot and Ankle Ability Measure, Patient Specific Functional Scale and amount of activity in the previous week. In addition, participants will be contacted by phone to obtain primary and secondary outcome measures at 12, 18, 24 and 30 weeks.

**Discussion:**

This article describes a single-blinded randomised controlled clinical trial that will utilise high quality methodologies in accordance with CONSORT guidelines. The results of this study will contribute to the limited knowledge regarding effective interventions for the management of ERLP.

**Trial registration:**

Australian New Zealand Clinical Trials Registry (ACTRN12613000914763)

**Electronic supplementary material:**

The online version of this article (doi:10.1186/1471-2474-15-328) contains supplementary material, which is available to authorized users.

## Background

Exercise related leg pain (ERLP) is a lower limb overuse injury characterised by pain located between the knee and ankle that occurs during activity. The condition encompasses the clinical and pathological features of several commonly used labels such as shin splints, medial tibial stress syndrome, periostitis, stress fractures, tendinopathies and compartment syndrome [1]. ERLP is particularly common in running populations with 13 to 20% of running injuries presenting to sports medicine clinics attributed to ERLP [1]. Incidence of ERLP has also been reported to be higher in females than males [2–6]. Significant burden is associated with the condition with 40-60% of runners reporting that ERLP caused a reduction in running or an interruption to training [7, 8]. Furthermore, the condition is associated with lengthy recovery periods in both runners and military personnel [9, 10]. Given the high incidence of the condition, subsequent interference with participation in physical activity and substantial recovery time, effective interventions for management of ERLP are required.

Several interventions have been described for the management of ERLP such as load management, low-energy laser treatment, stretching exercises, strengthening exercises, compression stockings, leg braces, pulsed electromagnetic fields, ice massage, ultrasound, iontophoresis, phonophoresis, and extracorporeal shockwave therapy. Whilst many of these interventions have been investigated in the literature, a recent systematic review [11] has reported a lack of evidence for an effective intervention for the condition. Despite the lack of research evidence, there appears to be consensus among clinical opinion pieces and review articles for the use of “relative rest” (load modification, alternative training), NSAIDS and ice in the acute phase of ERLP [1], and following this, the commencement of strengthening exercises for the calf and intrinsic foot muscles as well as more proximal muscles including the abdominals and gluteals with the aim of improving endurance and running mechanics [1, 12, 13]. To further develop the evidence base from expert opinion and anecdotal reports, Winters and colleagues [11] have suggested that it may be important to first understand the aetiological factors contributing to the condition.

A systematic review and meta-analysis by Newman et al. [14] suggests that the aetiology of ERLP is likely multifactorial. The authors reported evidence for several contributing factors: previous history of the condition (relative risk (RR) 3.74), female gender (RR 1.71), decreased running experience (standardised mean difference (SMD) -0.74), higher body mass index (SMD 0.24), history of orthotic use (RR 2.31) and >10 mm vertical displacement of the navicular from a subtalar neutral position to bodyweight supported stance (RR 1.99), commonly termed “navicular drop” [15]. Of these factors however only body mass index and navicular drop are potentially modifiable. Based on these findings, it is conceivable that interventions which reduce body mass index or reduce navicular drop may have the potential to influence pain and function in individuals with ERLP.

Navicular drop provides an indication of the vertical mobility of the midfoot, which can also be measured by dorsal arch height difference [15]. Interventions which have demonstrated effectiveness in reducing vertical mobility of the midfoot include taping and exercise. Anti-pronation taping techniques, such as the low-Dye and augmented low-Dye (ALD), have consistently demonstrated reductions in vertical mobility of the midfoot and increased medial longitudinal arch height immediately following application, during walking and jogging, and following 20mins of jogging [16, 17]. Of interest, the ALD taping technique has also demonstrated maintenance of an increase in medial longitudinal arch height following removal of tape after two weeks of continual wear [18]. Importantly, these biomechanical effects have been demonstrated in ERLP populations [17–19], however the efficacy of the ALD in the management of pain and function has not been evaluated. These anti-pronation taping techniques have traditionally in the past been applied using a rigid (inelastic) sports tape, however in recent times, and at least since the 2008 Olympic Games, there has been increasing popularity in the use of elastic tapes, such as Kinesio tape™ , Dynamic Tape™, KT-Tape™, SpiderTech™ and Cure Tape™ [20]. The reasons underlying the increasing popularity of elastic taping are not known. The elastic nature of the tape and alternate fabrication with more waterproof materials may lead to increased comfort. It is also conceivable that the elastic recoil in the tape may provide mechanical assistance or resistance (deceleration or acceleration) to movement, therein reducing the load demands on musculotendinous units while also permitting full range of motion to occur. Nevertheless despite their growing utility in the clinical setting, no study to date has evaluated the efficacy of an elastic taping technique in the management of ERLP.

Plantar intrinsic foot muscle training consisting of submaximal flexion of the metatarsophalangeal and proximal interphalangeal joints to raise medial longitudinal arch height has also demonstrated reductions in vertical midfoot mobility. Mulligan et al. [21] reported that a four week program consisting of three minutes daily of this plantar intrinsic foot muscle exercise, which progressed from sitting to bilateral and single leg stance, produced a reduction in navicular drop. This reduction in navicular drop was evident at conclusion of the program and was maintained another four weeks following cessation of the program. This training program, however, was evaluated in an asymptomatic population and has not been investigated in individuals with ERLP. In addition, there is evidence that during functional tasks motion of the foot is related to more proximal motion at the hip, suggesting that a whole limb approach to control midfoot mobility may be warranted. Specifically, increased navicular drop has been reported to be related to increased hip internal rotation during a single-leg squat [22] and moderate to strong correlations have been reported between frontal and transverse plane movements of the hip and frontal plane movement of the foot during walking [23, 24]. This evidence suggests that strengthening exercises for the hip abductor and external rotator muscles may also have the potential to influence foot mobility, however this has not been investigated.

The current lack of evidence for effective interventions for the management of ERLP warrants the conduct of further high quality randomised controlled trials. A reasonable rationale for the development of an effective intervention may be one based on current clinical expert opinion and addressing identified risk factors for the condition. As previously discussed, there appears to be a general consensus that advocates relative rest followed by strengthening exercise(s) for the management of ERLP. Whilst several risk factors for the condition have been identified, foot mobility is one of two modifiable factors. Anti-pronation taping and plantar intrinsic foot muscle training have demonstrated the ability to reduce foot mobility, however, no scientific study has evaluated the effectiveness of either intervention in the management of pain and function in ERLP.

## Methods

### Aim

The aim of this project is to compare the effectiveness of (i) lower limb muscle training alone, (ii) lower limb muscle training and rigid anti-pronation taping, and (iii) lower limb muscle training and elastic anti-pronation taping, for the management of ERLP.

### Study design

**Figure 1:**
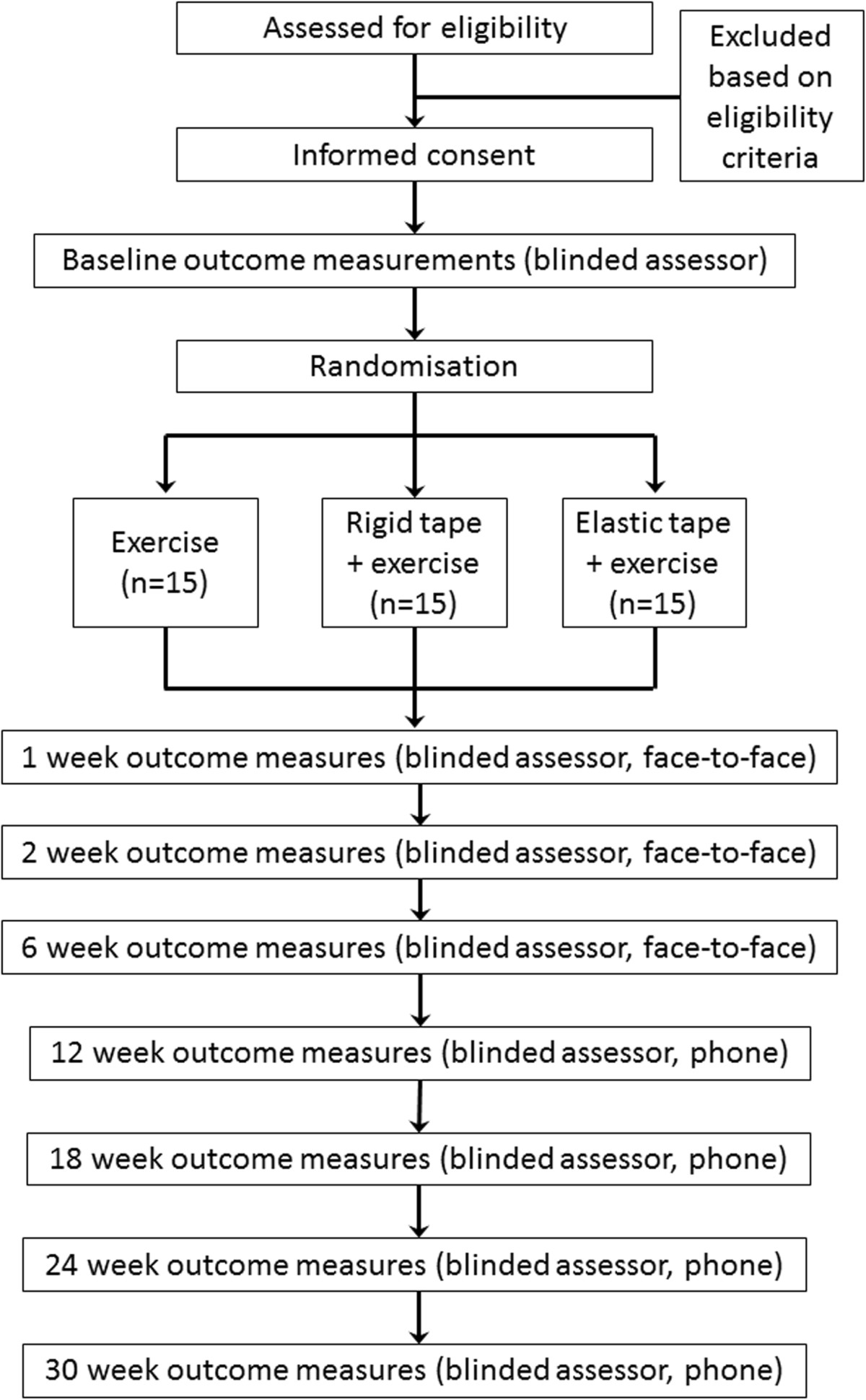
**Flow chart of participants through study.**

A randomised controlled clinical trial will be conducted in a community setting over a 12 month period. The Australian Catholic University Human Research Ethics Committee has granted ethical approval and all participants will provide informed written consent prior to commencement in the trial. An individual independent to the trial will perform the computer-generated randomisation sequence and preparation of envelopes containing the group allocation. The physiotherapist will communicate with participants regarding group allocation; due to the nature of the intervention it will not be possible to blind the treating physiotherapist or the participants. An assessor blinded to group allocation will perform outcome measurements at all time points. Data entry and statistical analysis will be performed by a blinded assessor. Figure 1 provides an overview of the study protocol.

### Eligibility criteria

Female volunteers will be eligible for participation in the trial on the basis of the following criteria: age 18 to 40 years, insidious onset of pain unrelated to any traumatic event, pain located between the knee and ankle of at least one month duration that is aggravated by weight bearing activities such as running, hopping or jumping, and worst pain over the previous week of at least 30 mm on a 100 mm visual analogue scale. Volunteers will be excluded from the trial in the presence of any of the following: a history of surgery to the lower limb, a cardiac condition, a known allergy to adhesive strapping tape, or symptoms of radiculopathy or other neurological involvement. Extensive previous exposure to either taping technique will also exclude volunteers from participation in order to prevent bias to one intervention.

### Recruitment of participants

Female volunteers will be recruited from paid advertisements in local community newspapers, supplemented by regular postings of advertisements on noticeboards within the University and wider community (e.g. gymnasiums, sporting clubs, health clinics). Each potential participant who responds to advertisements will undergo a screening process (via phone or email) to determine eligibility. Eligible volunteers will be provided with an information sheet thoroughly explaining the study and informed written consent to participate in the study will be obtained. Once eligibility has been determined and the informed consent process completed, participants will be assigned an identification number. Enrolled participants will attend a baseline testing session at which an assessor blinded to group allocation will obtain demographic information (age, weight, height), clinical characteristics of ERLP (nature, location, duration, onset, previous treatment), exercise history (intensity, duration and modality of current participation, and participation prior to the onset of ERLP), and perform outcome measurements. At the conclusion of baseline outcome measurements, the participant will be notified of group allocation by the treating physiotherapist and the intervention will commence.

### Randomisation

For group allocation, a computer-generated table of random numbers (in three blocks) will be used for the randomisation sequence. An individual not otherwise involved in the study will place the randomisation sequence in a series of consecutively numbered opaque envelopes, and sign across the seal of the envelope. Allocation will be concealed from the outcome assessor for the duration of the trial.

### Intervention

The interventions will be performed by one of two registered physiotherapists (Physiotherapy Board of Australia), each with at least 10 years of experience in musculoskeletal physiotherapy. In addition, both physiotherapists will have completed two comprehensive training sessions together for explanation and discussion of the standardised intervention protocol for the trial. A detailed written protocol outlining trial procedures will be provided to treating physiotherapists. This will include standardised instructions for participant advice, application of both the rigid and elastic taping techniques, prescription of the lower limb muscle training exercises, and recording of treatment. Over a six week intervention period participants will receive one of three treatments: (i) lower limb muscle training alone, (ii) combined intervention of rigid tape and lower limb muscle training, (iii) combined intervention of elastic tape and lower limb muscle training.

#### Lower limb muscle training alone

Participants in this group will receive advice and education and will be prescribed an exercise program to perform at home. At the completion of the baseline outcome measurements, the physiotherapist will provide a standardised information sheet with advice and education regarding ERLP, as well as load management. Participants will be advised not to start any new activities or treatments for the duration of the study, encouraged to continue to wear current footwear (including orthotic use) and participate in activities that do not provoke symptoms and to avoid activities that aggravate symptoms either during or following the activity; as treatment progresses, this may include a return to participation in activities that previously aggravated symptoms.

**Figure 2:**
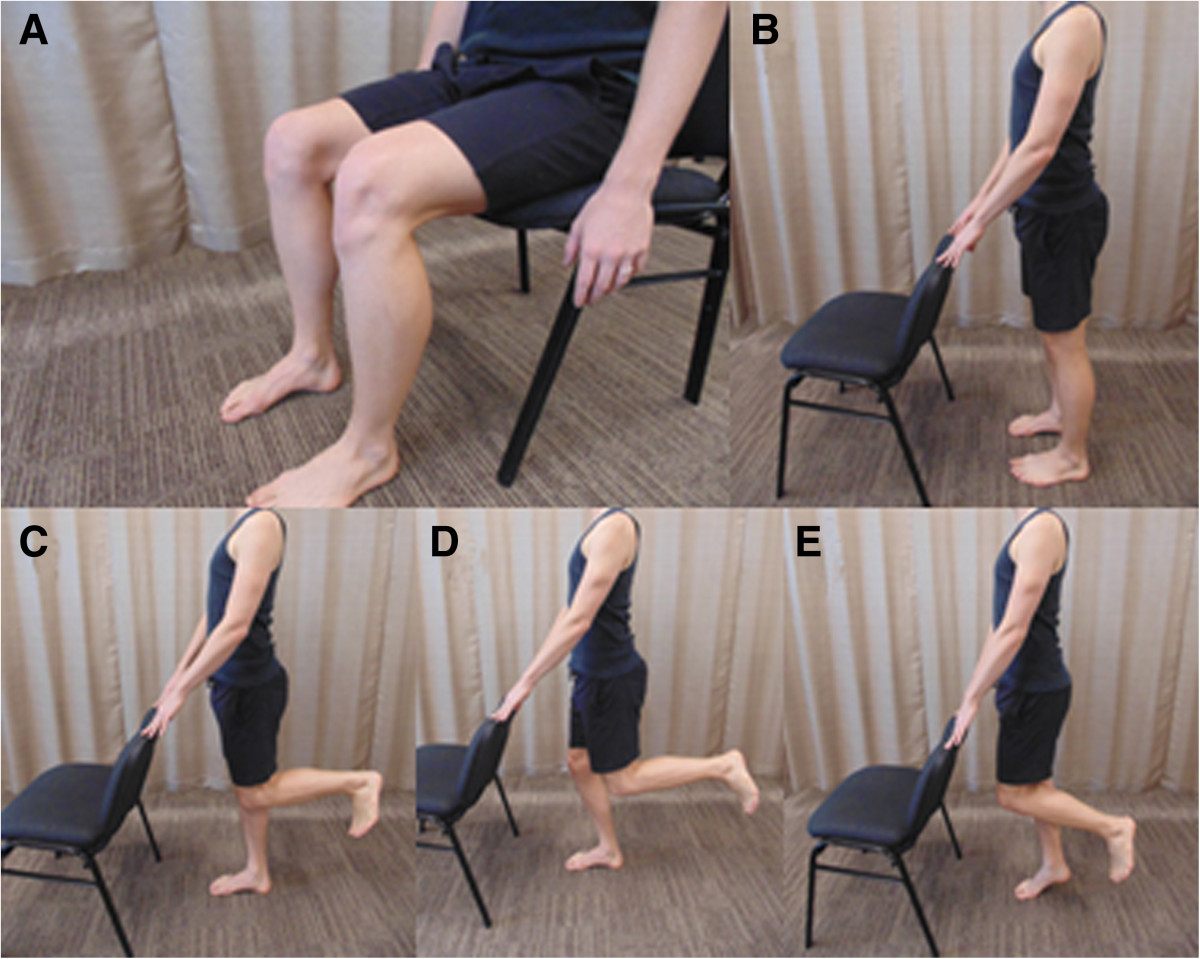
**Plantar intrinsic foot muscle training.** Plantar intrinsic foot muscle training will be performed as described by Mulligan et al. [21]. Active intrinsic foot muscle exercises may be commenced in a non-weight bearing sitting position **(A)**. The patient is instructed to increase the medial longitudinal arch by gently supinating the foot and approximating the head of the first metatarsal towards the heel, without flexing the toes. This position should be held for 5 seconds and then slowly released back to a relaxed state. Patients should aim to perform up to 3 minutes of this exercise daily. Exercises can then be progressed to functional weight bearing positions, for example double-limb stance **(B)** and single-limb stance **(C)**. For further progression, a small knee bend **(D)** and/or heel raise **(E)** can be added to these functional positions.

**Figure 3:**
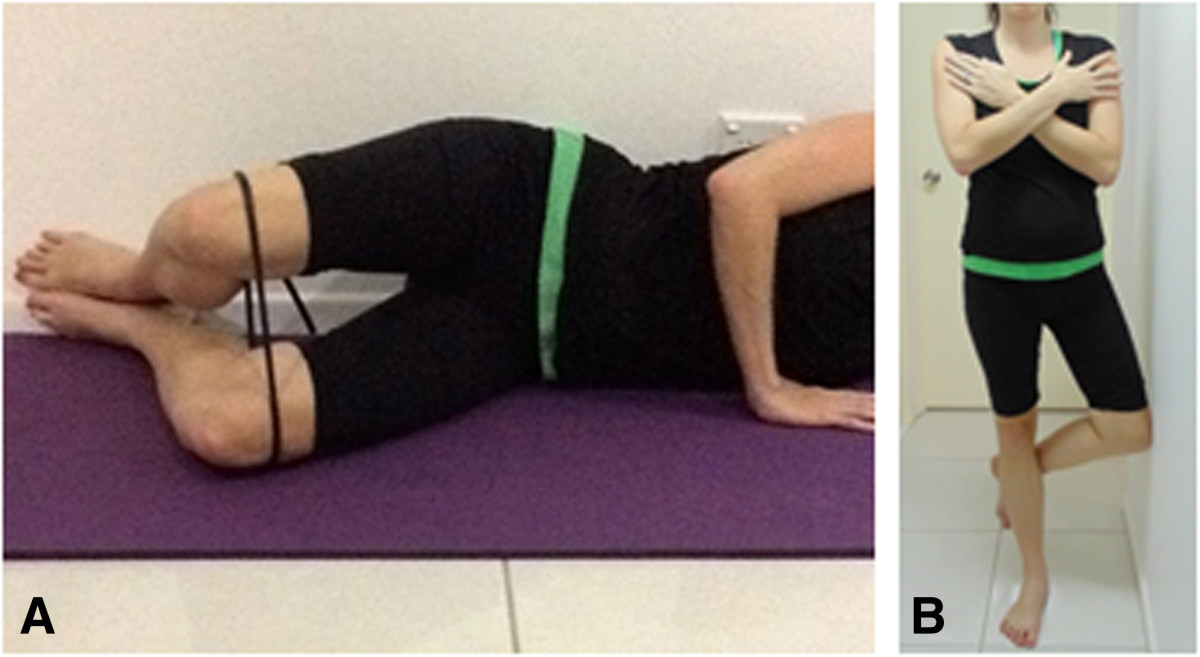
**Hip abductor and external rotator muscle training.** For the non-weight bearing and weight bearing exercises, the patient will perform a 20 second hold and aim to perform 3 repetitions, daily. The non-weight bearing exercise **(A)** will be performed in side-lying with the treatment limb uppermost and both limb flexed to 45° at the hip and 90° at the knee. Ideally the patient’s back and plantar surface of the foot are placed against a wall for control of position and movement. The patient raises the top limb off the other, such that the hip is in ~30° abduction/external rotation before returning to the starting position. The weight bearing exercise **(B)** is performed with the patient standing side-on to a wall with body rotated slightly to face into the wall. The leg closest to the wall is flexed at the knee so that the foot is off the ground, and the hip is in neutral flexion/extension. The foot of the leg closest to the wall is tucked behind the knee of the outer leg. The standing leg knee should be bent slightly. The patient is instructed to squeeze the bottom together and hold this throughout the movement, then turn the knee of the standing leg out without moving the foot or pelvis. The bent leg is held against the wall for balance only.

At the end of week 1, the participant will attend a thirty minute physiotherapy session where advice and education information will be reviewed with the patient and training in the exercise program will commence. The exercise program will include two exercises: (i) the plantar intrinsic foot muscle exercise [21] (Figure 2), and (ii) hip abductor and external rotator muscle exercise (Figure 3). The difficulty of both exercises can be increased from non-weightbearing, to double-limb weightbearing and then single-limb weightbearing (Figures 2 and 3). A rating scale to assess the intrinsic foot muscles [21], based on unsteadiness of navicular height during single-limb stance, will be used to decide the difficulty of the exercises prescribed at the first session: non-weightbearing exercises will be prescribed for a rating of poor stability; double limb weightbearing exercises will be prescribed for a rating of fair stability, and single-limb weightbearing will prescribed for a rating of good stability. Participants will be instructed how to perform the exercises at each level of difficulty, and will be encouraged to increase the difficulty of the exercises when full repetitions are achieved (3 minutes for intrinsic foot muscles, 3 × 20 second holds for gluteal muscles). Participants will be instructed to perform the exercises daily and record the exercises they completed and their difficulty level in a training diary for the duration of week 2 of the trial. At the end of week 2, the participant will attend a thirty minute physiotherapy session to reinforce advice and education, review the exercise program and progress exercises as able. Participants will be instructed to perform the exercises daily and record the exercises they completed and their difficulty level in a training diary for a further 4 weeks. To check exercise progression and encourage compliance, the treating physiotherapist will contact participants weekly by telephone. At conclusion of the 6 week intervention, participants will be encouraged to continue prescribed exercises and load management strategies and this self management will be recorded in follow-up questionnaires at 12, 18, 24 and 30 weeks.

#### Combined rigid anti-pronation taping and lower limb muscle training

**Figure 4:**
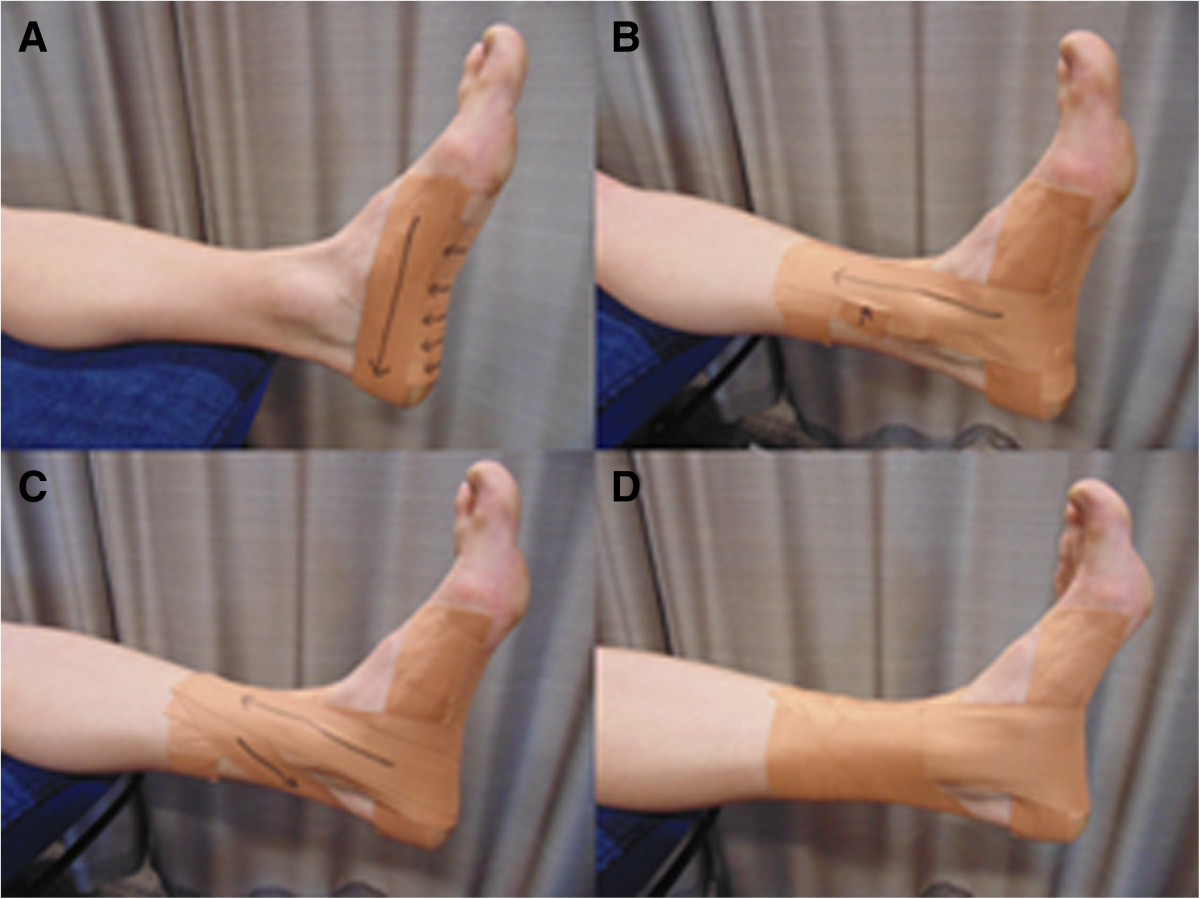
**Rigid anti-pronation taping technique (ALD).** The ALD technique will be applied following guidelines previously described [25]. The ALD comprises of a low-Dye technique **(A)** with the addition of three reverse-6 techniques **(B)** and two calcaneal sling techniques **(C)** that are anchored to the lower third of the leg. The technique is completed with circumferential lock off strips from the proximal anchor to just above the malleoli **(D)**.

Participants will receive advice and education, be prescribed an exercise program and wear rigid ALD taping [25] (Figure 4). At the completion of the baseline outcome measurements, the physiotherapist will provide a standardised information sheet, as described above. Following this, ALD taping will be applied using a rigid sports tape (38 mm zinc oxide adhesive, Leukosport BDF™) and the appropriate skin checks made and warnings given. Participants will be re-taped half way through week 1; no advice or education regarding the exercises will occur during this session. Participants will be instructed to remove the tape and any tape residue the night prior to attending the testing session. At the end of week 1, the participant will attend a thirty minute physiotherapy session where advice and education will be reinforced, the ALD tape re-applied and training in the exercise program commenced. The exercise program will include the same two exercises as described above: (i) intrinsic foot muscle exercise, and (ii) hip abductor and external rotator muscle exercise. Participants will be instructed to perform the exercises daily and record their performances in a training diary for the duration of week 2 of the trial. Participants will be re-taped half way through week 2; no advice or education regarding the exercises will occur during this session. Participants will be instructed to remove the tape and any tape residue the night prior to attending the testing session. At the end of week 2, the participant will attend a thirty minute physiotherapy session to reinforce advice and education, review the exercise program and progress exercises as able. Participants will be instructed to perform the exercises daily and record in a training diary for a further 4 weeks. To check progress and encourage compliance, the treating physiotherapist will contact participants weekly by telephone. No taping will be applied during these final 4 weeks of the intervention.

#### Elastic anti-pronation taping and lower limb muscle training

**Figure 5:**
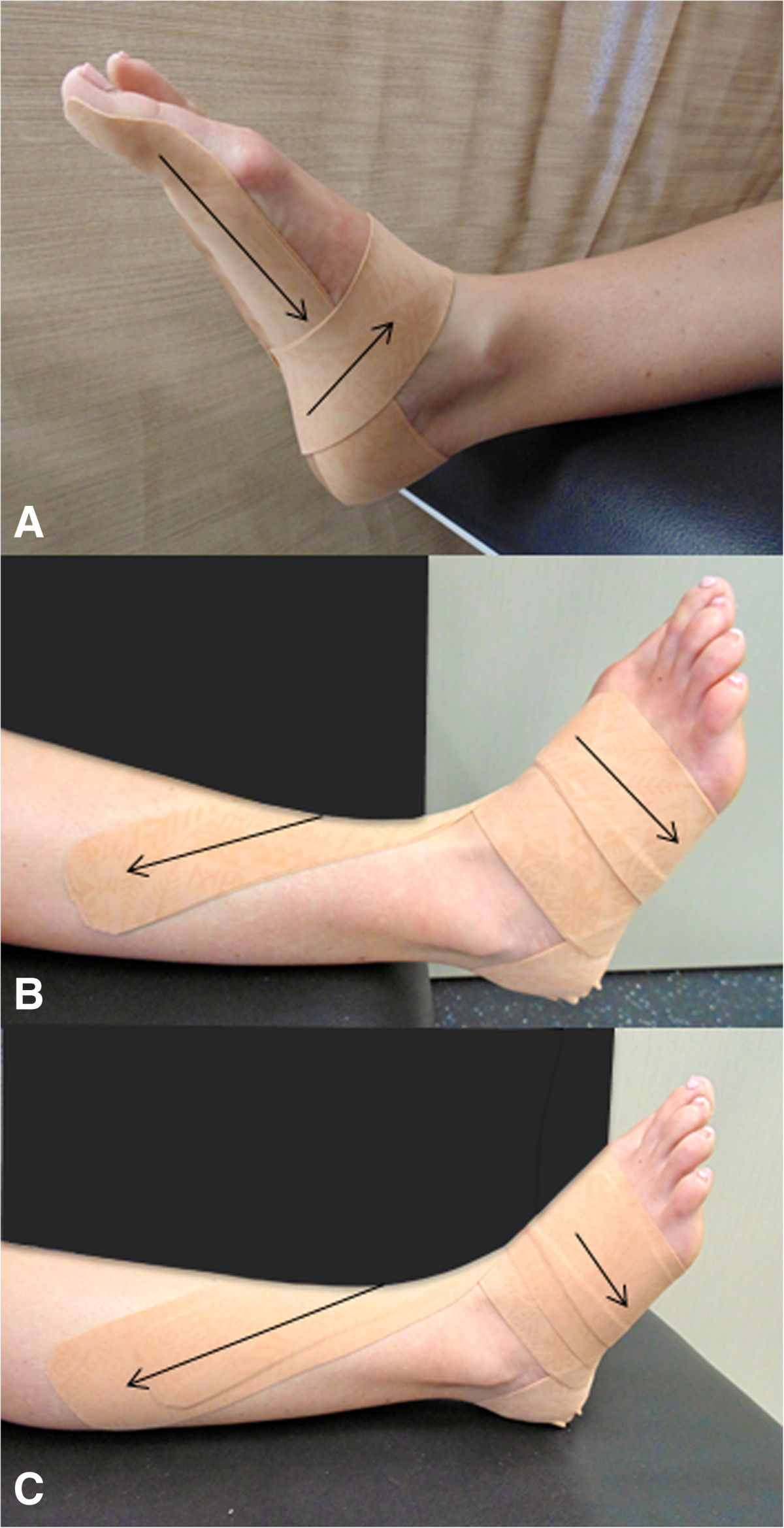
**Elastic anti-pronation taping technique.** The elastic anti-pronation taping technique will be applied following Dynamic Tape™ guidelines for the plantar fascia and anti-pronation techniques [26]. For each technique a double layer of 50 mm Dynamic Tape™ will be used. The plantar fascia technique **(A)** is applied by cutting out a small wedge from one end of the double layered tape to allow it to conform to the proximal phalanx of the first toe. With the foot and ankle in full plantarflexion, inversion, adduction and flexion of the first toe, the tape is applied from the plantar surface of the first toe, along the medial plantar aspect of the foot, onto the medial aspect of the calcaneus, around the calcaneus to cross the lateral aspect obliquely, continuing under the plantar aspect of the foot to emerge under the navicular and finally lifting the navicular to anchor the tape on the dorsum of the foot. The anti-pronation technique **(B)** is then applied with the foot and ankle in full dorsiflexion, inversion, and adduction. The double layered tape is applied from the plantar surface of the foot between the first and second metatarsals, courses over the dorsum of the foot from medial to lateral, under the plantar aspect of the foot on a diagonal course to the navicular, lifting the navicular and coursing across the anterior talocural joint to anchor on the lateral aspect of the mid-shin region. The anti-pronation technique is then repeated with one layer of 75 mm Dynamic Tape™ **(C)**.

Participants will receive advice and education, be prescribed an exercise program and wear an elastic anti-pronation taping [26] (Figure 5). At the completion of the baseline outcome measurements, the physiotherapist will provide advice and education regarding the condition and load management. Following this, elastic anti-pronation taping will be applied using an elastic tape (50 mm & 75 mm, Beige Dynamic Tape™) and the appropriate skin checks made and warnings given. Participants will be re-taped half way through week 1; no advice or education regarding the exercises will occur during this session. Participants will be instructed to remove the tape and any tape residue the night prior to attending the testing session. At the end of week 1, the participant will attend a thirty minute physiotherapy session where advice and education will be reinforced, the elastic tape re-applied and training in the exercise program will commence. The exercise program will include the same two exercises as described above: (i) intrinsic foot muscle exercise, and (ii) hip abductor and external rotator muscle exercise. Participants will be instructed to perform the exercises daily and record their performances in a training diary for the duration of week 2 of the trial. Participants will be re-taped half way through week 2; no advice or education regarding the exercises will occur during this session. Participants will be instructed to remove the tape and any tape residue the night prior to attending the testing session. At the end of week 2, the participant will attend a thirty minute physiotherapy session to reinforce advice and education, review the exercise program and progress exercises as able. Participants will be instructed to perform the exercises daily and record in a training diary for a further 4 weeks. To check progress and encourage compliance, the treating physiotherapist will contact participants weekly by telephone. No tape will be applied during these final 4 weeks of the intervention.

### Outcome measurements

A blinded assessor will perform tests of foot mobility and other outcome measurements at baseline and then at follow-up sessions occurring at 1, 2, and 6 weeks. Participants will also be contacted by email or phone to obtain questionnaire outcome measurements only at 12, 18, 24 and 30 weeks. At the completion of the test sessions, assessor blinding will be evaluated via a questionnaire.

Biomechanical effectiveness of the intervention will be evaluated by measuring vertical mobility of the midfoot. These measures will be performed at baseline and then at follow-up sessions occurring at 1, 2, and 6 weeks. In addition to these measurements by the assessor, the treating physiotherapist will also obtain a measurement of foot mobility before and after each application of tape to ensure consistency of application of the intervention. Measurement of vertical mobility of the midfoot will be performed using two custom made platforms and modified callipers with the participant in two positions (i) standing on both feet with equal weightbearing and, (ii) sitting with legs relaxed over the edge of a plinth. Dorsal arch height difference will be calculated by subtracting the dorsal arch height in standing from the dorsal arch height in non-weight bearing. This measurement has been described in detail previously and demonstrates high inter- and intra-rater reliability [15].

#### Visual Analogue Scale

Usual and worst pain over the preceding week will be obtained using two separate 100 mm visual analogue scales (VAS). The reliability and validity of the VAS is well established [27]. Each VAS will consist of a 100 mm horizontal line with the text ‘no pain’ at the 0 mm mark and ‘worst pain imaginable’ at the 100 mm mark. Participants will be instructed to place a single vertical mark on the horizontal line that represents the level of their pain.

#### Foot and Ankle Ability Measure

Function will be measured using the Foot and Ankle Ability Measure (FAAM) which provides an indication of participation in usual activities [28]. The FAAM is a set of eight (8) items that are each scored by the participant from 4 to 0, with a score of 4 indicating no difficulty with the functional task and 0 indicating that the participant is unable to perform the task. Thus, the greater the FAAM score, the higher the level of function. The validity, reliability and responsiveness of the FAAM has been previously demonstrated [28].

#### Patient Specific Functional Scale

The Patient Specific Functional Scale (PSFS) provides a method for eliciting, measuring and recording descriptions of patients’ disabilities that are most relevant to them. Participants will be asked to identify up to five (5) important activities that they are having difficulty with or are unable to perform. The current level of difficulty associated with each activity is then marked on an 11-point scale, where 0 is ‘unable to perform activity’ and 10 is ‘able to perform activity at the same level as before the injury or problem’. This scale has been shown to have excellent test-retest reliability, sensitivity to change, and validity [29].

#### Global perceived effect

Global perceived effect will be measured with a 6-point Likert scale with categories: completely recovered, much improved, improved, no change, worse and much worse. Such retrospective assessment scales have been shown to be more sensitive to change and more correlated with patient’s satisfaction with change than serial assessment scales that include a baseline measurement, such as the VAS [30]. This outcome measure will be performed at each of the follow-up sessions, but not at baseline.

#### Amount of activity performed in the previous week

The physical activity level of participants will be quantified using a physical activity questionnaire which has previously demonstrated moderate to high reliability [31]. The participant will record the amount of time spent in occupational, household, and leisure activities of ‘moderate’, ‘hard’ and ‘very hard’ intensities during the previous week. The total time for each intensity level of activity is then multiplied by the metabolic equivalents of the activities and summed to give an overall caloric output. This can then be standardised to body weight to allow comparison between and within individuals over time.

A further questionnaire regarding any adverse responses and perception of comfort of the taping interventions, as well as whether participants have sought any treatment outside the study, will be administered at follow-up sessions at 1, 2 and 6 weeks.

### Sample size considerations

Sample size calculations [32] indicate that 10 participants per group will be sufficient to detect (i) a clinically significant improvement of 20 mm in pain severity [33] based on a standard deviation of 12.7 mm [34], power of 0.80 and alpha level 0.05 and (ii) a clinically significant improvement of 9 points [28] on the FAAM based on a standard deviation of 26.7 [28], power of 0.80 and alpha level of 0.05. To account for potential dropout, 15 participants per group will be recruited, with a total sample size of 45.

### Planned statistical analysis

Data processing, data entry and data analysis will be performed by an assessor who is blinded to group allocation. The primary analysis of the data will be performed on an intention-to-treat basis. SPSS software (version 21.0) will be used for statistical procedures. Demographic data, clinical characteristics of pain and physical activity level at baseline will be examined for comparability across the three intervention groups. To evaluate the effect of the interventions on pain and function (VAS, FAAM, PSFS, Physical activity level) a two-way repeated measure analysis of variance with between subject factor of intervention group and within subject factor of test session, will be performed. Global perceived effect will be dichotomised to either success (completely recovered, much improved, improved) or no success (no change, worse, much worse) and expressed as relative risk reduction and numbers needed to treat. For all statistical procedures age, weight and height will be included as covariates and alpha level will be set at 0.05.

## Discussion

This study will be the first randomised controlled trial to evaluate the effectiveness of anti-pronation taping and lower limb muscle training for ERLP. As there is currently a lack of evidence for effective interventions for ERLP [11], the results of this study will provide evidence to inform healthcare providers in the management of the condition. For example, if this study identifies one intervention to be superior to the others, this intervention can be recommended in preference to others. Alternatively, if no differences are identified between the three intervention groups, then taping would be considered to have no additional benefit over exercise alone and it would therefore be recommended that clinicians could focus on exercise alone rather than additional taping. An important feature of this study is the inclusion of a follow-up at 12, 18, 24 and 30 weeks. Accordingly, this study will provide an evaluation of both the short and long term effectiveness of an intervention for ERLP.

The three intervention groups evaluated in this study were selected to target a reported risk factor for the condition (increased foot mobility) as well as to reflect clinical practice. Clinical opinion pieces and review articles advocate the reduction of pain through “relative rest” (load modification, alternative training), NSAIDS and ice in the acute phase of ERLP, and following this, the commencement of strengthening exercises [1, 12, 13]. Therefore, we selected a lower limb muscle training intervention which consisted of advice and education on ERLP and load management, followed by strengthening exercises. For comparison, we included a combined intervention of taping and lower limb muscle training. This was selected to reflect a tissue stress model approach to management whereby the short term use of external devices (such as taping) is advocated to alleviate tissue stress and then followed by conventional physical therapy modalities such as muscle strengthening exercises [35, 36].

The rigid anti-pronation taping technique (ALD) selected for this randomised controlled trial is a well-established and described technique which has previously demonstrated biomechanical effectiveness in ERLP populations. A strength of this randomised controlled trial is the inclusion of a comparative elastic anti-pronation taping technique. Despite a rapid uptake of these tapes clinically, there is limited evidence as to their biomechanical or clinical efficacy, and specifically, no study has investigated an elastic taping technique in an ERLP population. In addition to evaluating the clinical efficacy of the rigid and elastic anti-pronation taping techniques, this study may also enable some comparisons to be made between them, including incidence of adverse responses and perceptions of comfort.

The lower limb muscle training selected for this randomised controlled trial includes two exercises; one aimed at the plantar intrinsic foot muscle and one aimed at the external rotator and abductor muscles of the hip. Plantar intrinsic foot muscle training was included in this study as previous evidence has reported effectiveness to influence vertical mobility of the midfoot [21], a factor that has been prospectively identified as a contributing to the development of ERLP [14]. Whilst there is no study to date that has investigated the use of hip external rotator and abductor muscle training on foot posture or mobility, there is theoretical evidence that supports the rationale for its inclusion. Aligning with a kinetic chain approach to lower limb function, frontal and transverse plane hip motion has been shown to be strongly correlated to frontal plane foot motion during walking [23, 24], and increased navicular drop has also been shown to be related to increased hip internal rotation during a single-leg squat [22]. This is supported by the finding of altered proximal control of gait, specifically the gluteus medius (abductor and external rotator of the hip), in individuals with ERLP [34]. The non-weight bearing hip rotation exercise was selected because it is a relatively simpler task with less body segments to control. It is also a commonly prescribed exercise for gluteus medius clinically and previous fine-wire investigations have reported higher gluteal-to-TFL muscle activation compared with other hip exercises [37]. The weight bearing exercise progression was selected to increase complexity and to facilitate relevance to the functional tasks of walking and running. Specifically it was chosen as it closely resembles the midstance position of these activities and has been used in previous studies to target gluteal strengthening in other lower limb overuse conditions [38, 39].

This randomised controlled trial is registered with the Australian New Zealand Clinical Trials Registry (ACTRN12613000914763) and will comply with the CONSORT statement. Previous randomised controlled trials in ERLP populations have exhibited high risk of bias [11] and therefore this study has been designed with consideration to minimise these limitations. Specifically this study design incorporates a random sequence generation and concealed allocation to minimise selection bias, blinding of the outcome assessor to group allocation to minimise detection bias and blinding of the investigators responsible for data processing and statistical analysis to minimise possible bias associated with their anticipated outcomes. Unfortunately, due to the nature of the interventions, blinding of participants and the treating physiotherapist to prevent performance bias is not possible. Finally, to ensure high methodological quality, outcome measures with established reliability and validity have been selected. A limitation of the methodology is the recruitment of female participants of less than 40 years of age; therefore the results of this study may not be generalizable to male and/or older populations with ERLP. This limitation, however, should be considered in light of evidence that female gender has been reported as a risk factor in ERLP [14] and higher incidence of ERLP has been reported in both females and the 18–40 years age group [2–6, 40].

## Authors’ original submitted files for images

Below are the links to the authors’ original submitted files for images. Authors’ original file for figure 1 Authors’ original file for figure 2 Authors’ original file for figure 3 Authors’ original file for figure 4 Authors’ original file for figure 5

## Abbreviations

ALD: Augmented low-Dye
ERLP: Exercise related leg pain
FAAM: Foot and Ankle Ability Measure
PSFS: Patient Specific Functional Scale
RR: Relative risk
SMD: Standardised mean difference
VAS: Visual analogue scale.
