# Supplementary figures and images for: A comparison of rigid tape and exercise, elastic tape and exercise and exercise alone on pain and lower limb function in individuals with exercise related leg pain: a randomised controlled trial
Source: BMC Musculoskelet Disord. 2014 Oct 2;15:328. doi: 10.1186/1471-2474-15-328 (PMC4201713; doi:10.1186/1471-2474-15-328)

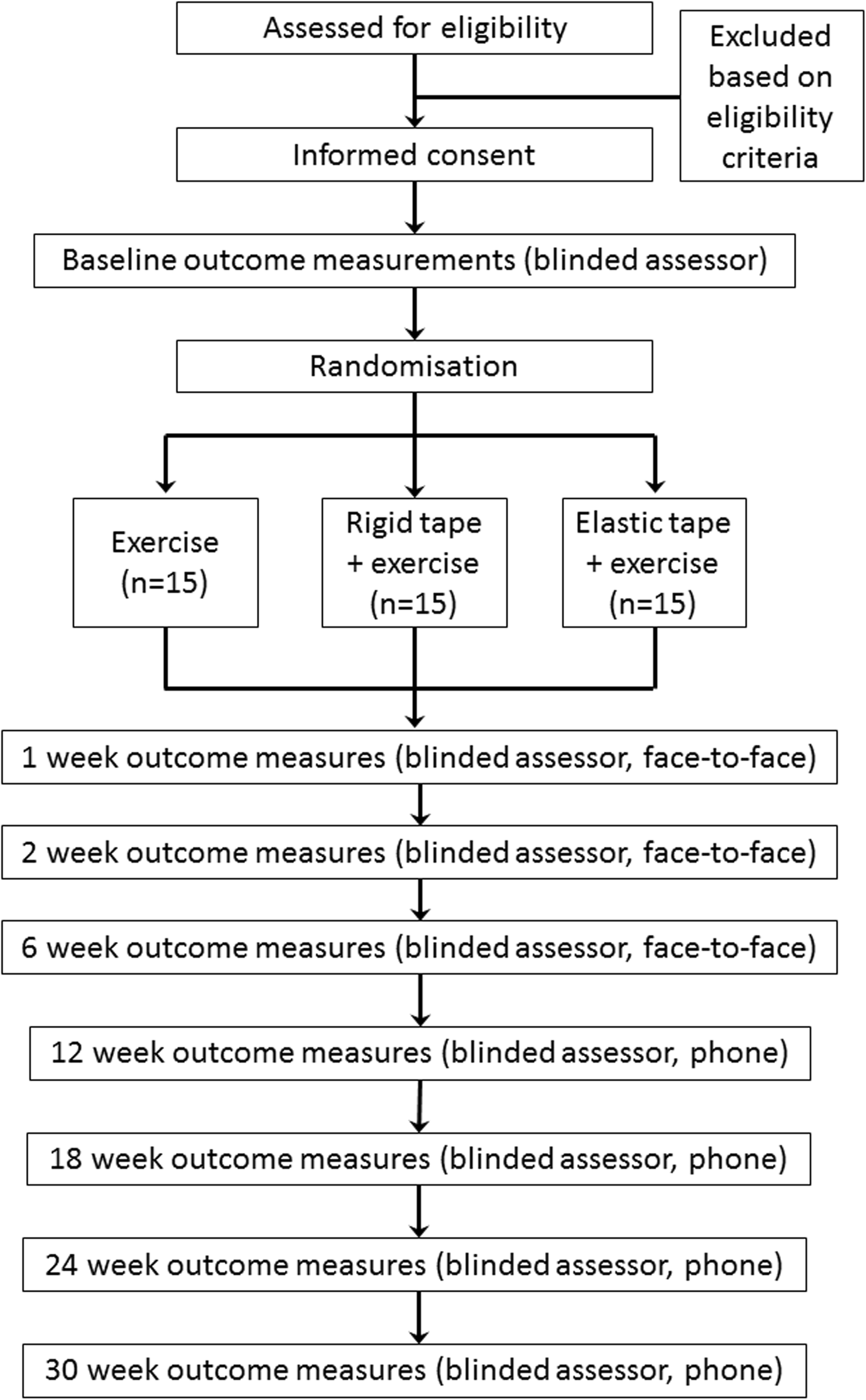

Supplement: Supplementary file 1 — Authors’ original file for figure 1 [file 12891_2014_2278_MOESM1_ESM.tif]

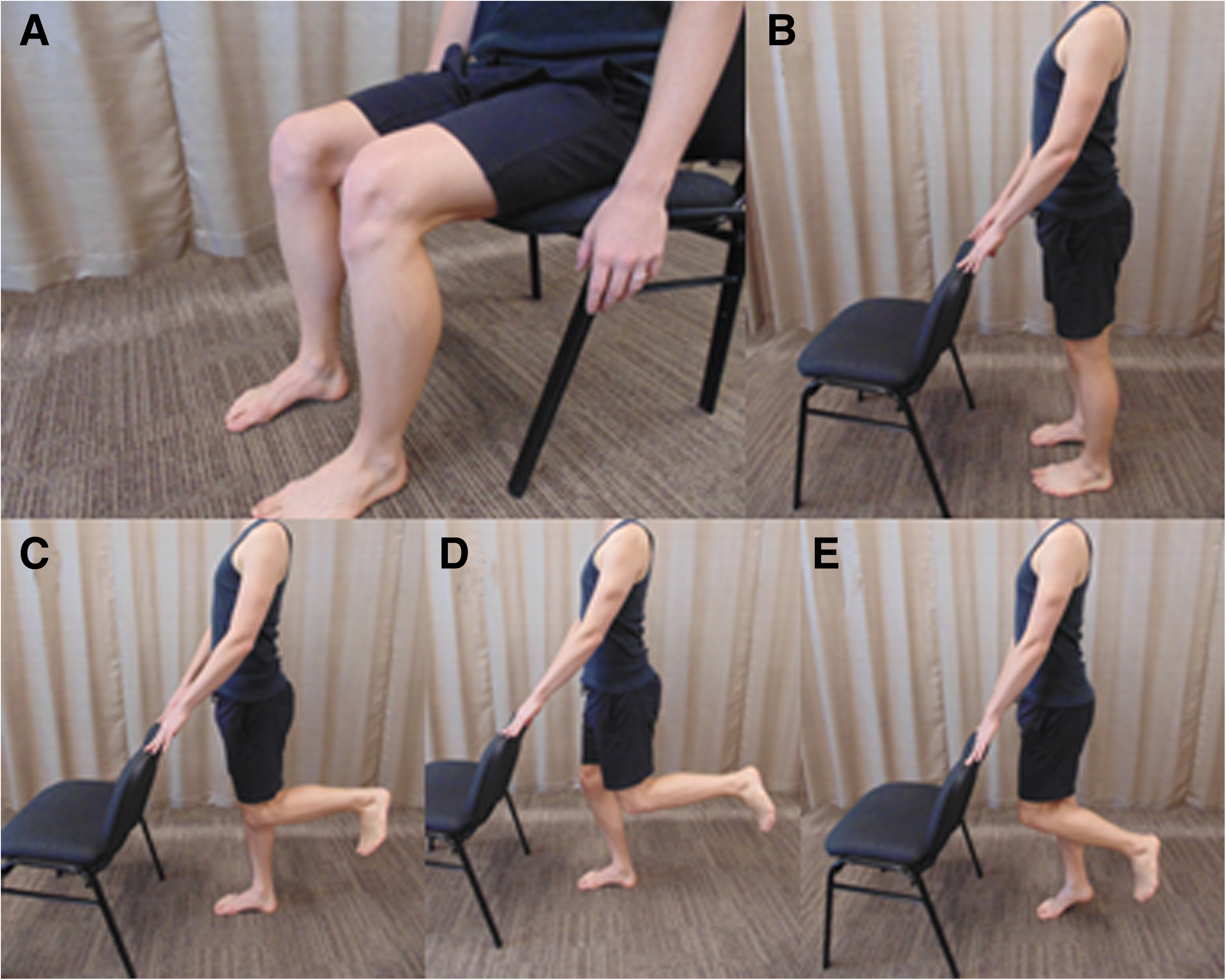

Supplement: Supplementary file 2 — Authors’ original file for figure 2 [file 12891_2014_2278_MOESM2_ESM.tif]

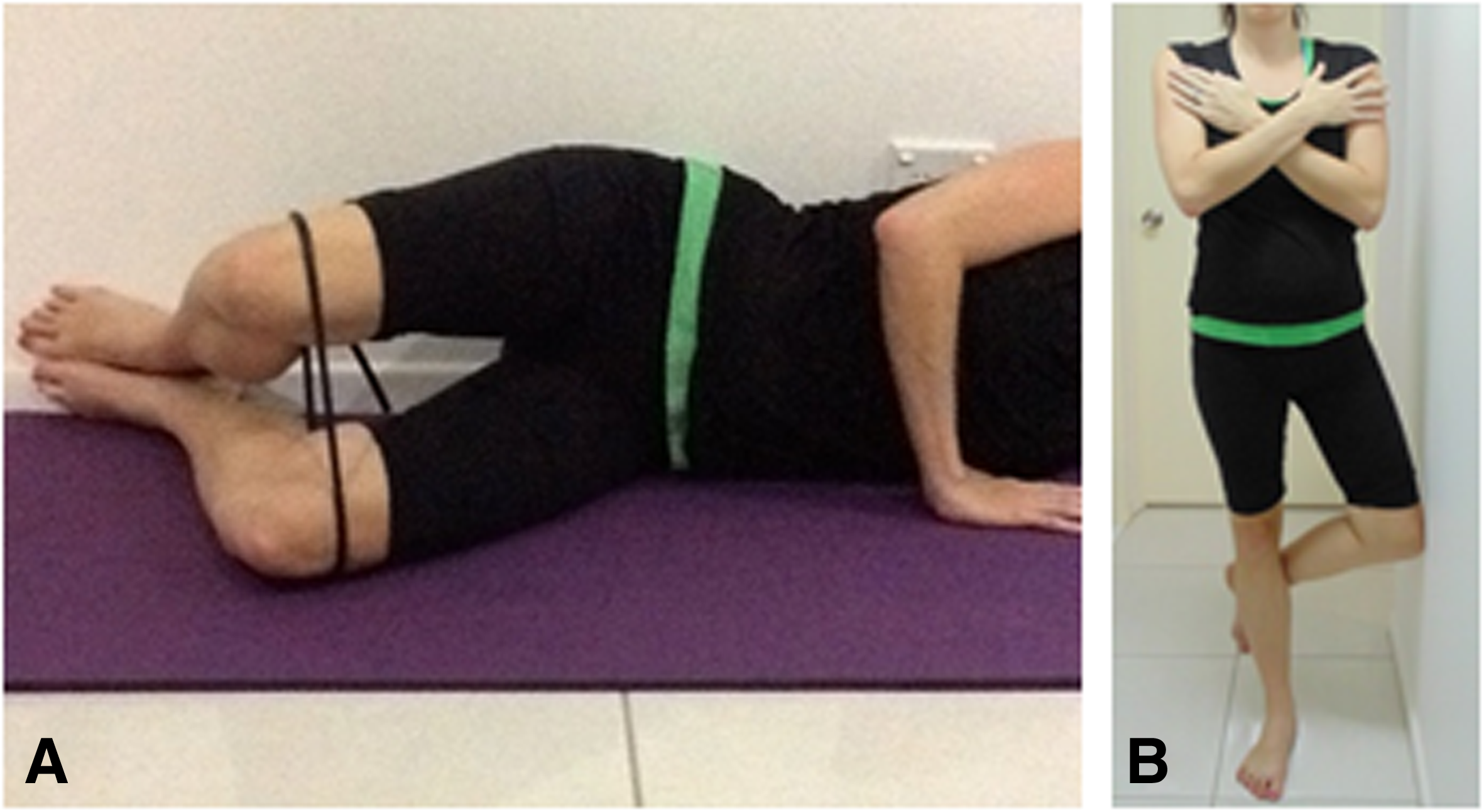

Supplement: Supplementary file 3 — Authors’ original file for figure 3 [file 12891_2014_2278_MOESM3_ESM.tif]

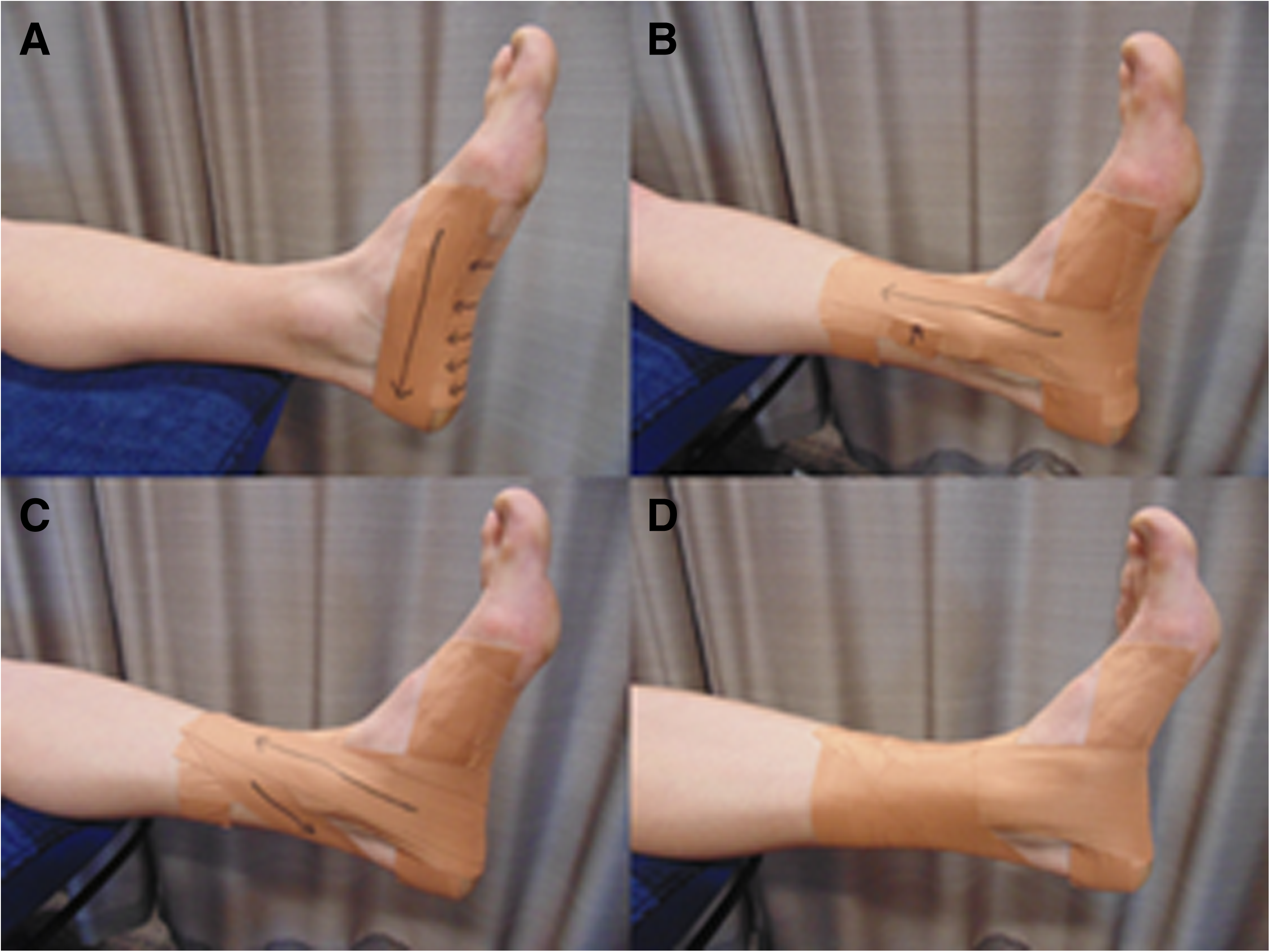

Supplement: Supplementary file 4 — Authors’ original file for figure 4 [file 12891_2014_2278_MOESM4_ESM.tif]

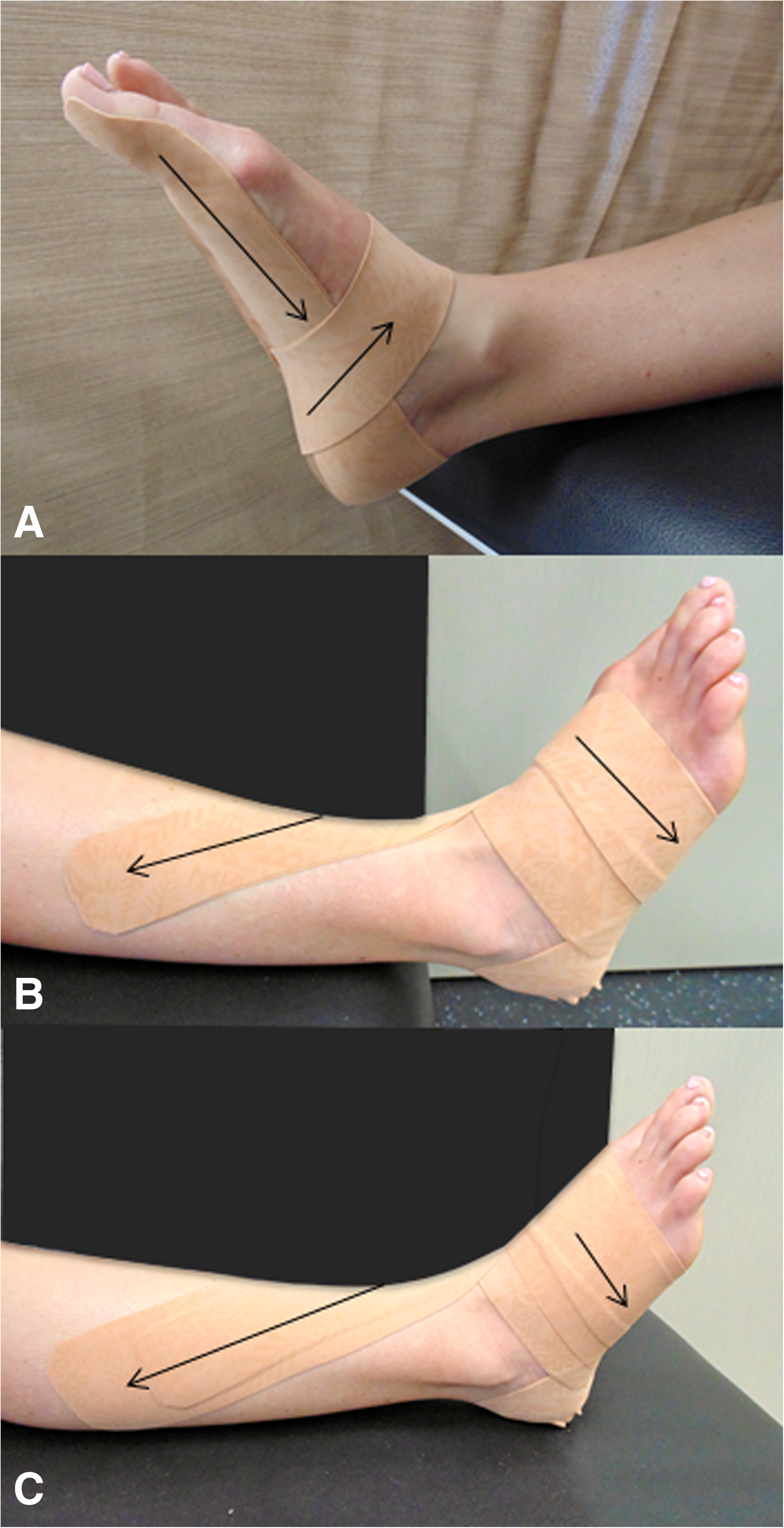

Supplement: Supplementary file 5 — Authors’ original file for figure 5 [file 12891_2014_2278_MOESM5_ESM.tif]
